# Dosimetric performance of the new high‐definition multileaf collimator for intracranial stereotactic radiosurgery

**DOI:** 10.1120/jacmp.v11i3.3040

**Published:** 2010-06-21

**Authors:** Anees Dhabaan, Eric Elder, Eduard Schreibmann, Ian Crocker, Walter J. Curran, Nelson M. Oyesiku, Hui‐Kuo Shu, Tim Fox

**Affiliations:** ^1^ Emory University Department of Radiation Oncology Atlanta GA 30322 USA

**Keywords:** Conformity Index, radiosurgery, high‐definition MLC, normal tissue sparing, Novalis TX

## Abstract

The objective was to evaluate the performance of a high‐definition multileaf collimator (MLC) of 2.5 mm leaf width (${\text{MLC}}_{2.5}$) and compare to standard 5 mm leaf width MLC (${\text{MLC}}_{5}$) for the treatment of intracranial lesions using dynamic conformal arcs (DCA) technique with a dedicated radiosurgery linear accelerator. Simulated cases of spherical targets were created to study solely the effect of target volume size on the performance of the two MLC systems independent of target shape complexity. In addition, 43 patients previously treated for intracranial lesions in our institution were retrospectively planned using DCA technique with ${\text{MLC}}_{2.5}$ and ${\text{MLC}}_{5}$ systems. The gross tumor volume ranged from 0.07 to $40.57\;{\text{cm}}^{3}$ with an average volume of $5.9\;{\text{cm}}^{3}$. All treatment parameters were kept the same for both MLC‐based plans. The plan evaluation was performed using figures of merits (FOM) for a rapid and objective assessment on the quality of the two treatment plans for ${\text{MLC}}_{2.5}$ and ${\text{MLC}}_{5}$. The prescription isodose surface was selected as the greatest isodose surface covering ≥95% of the target volume and delivering 95% of the prescription dose to 99% of target volume. A Conformity Index (CI) and conformity distance index (CDI) were used to quantifying the dose conformity to a target volume. To assess normal tissue sparing, a normal tissue difference (NTD) was defined as the difference between the volume of normal tissue receiving a certain dose utilizing ${\text{MLC}}_{5}$ and the volume receiving the same dose using ${\text{MLC}}_{2.5}$. The CI and normal tissue sparing for the simulated spherical targets were better with the ${\text{MLC}}_{2.5}$ as compared to ${\text{MLC}}_{5}$. For the clinical patients, the CI and CDI results indicated that the ${\text{MLC}}_{2.5}$ provides better treatment conformity than ${\text{MLC}}_{5}$ even at large target volumes. The CI's range was 1.15 to 2.44 with a median of 1.59 for ${\text{MLC}}_{2.5}$ compared to 1.60–2.85 with a median of 1.71 for ${\text{MLC}}_{5}$. Improved normal tissue sparing was also observed for ${\text{MLC}}_{2.5}$ over ${\text{MLC}}_{5}$, with the NTD always positive, indicating improvement, and ranging from 0.1 to 8.3 for normal tissue receiving 50% (${\text{NTV}}_{50}$), 70% (${\text{NTV}}_{70}$) and 90% (${\text{NTV}}_{90}$) of the prescription dose. The ${\text{MLC}}_{2.5}$ has a dosimetric advantage over the ${\text{MLC}}_{5}$ in Linac‐based radiosurgery using DCA method for intracranial lesions, both in treatment conformity and normal tissue sparing when target shape complexity increases.

PACS number: 87.56J‐, 87.56 jk

## I. INTRODUCTION

Stereotactic radiosurgery (SRS) is the process of delivering a high dose of external beam radiation to a small intracranial target in a single fraction using ${\;}^{60}\text{Co}$ sources, medical linear accelerators, or charged particle beams guided by an external frame system. Collimated radiation beams are precisely positioned and focused onto a target within the brain to deliver a high dose of localized radiation. An advantage of SRS is the prevention of damage to surrounding healthy tissue because of the steep dose gradient around the target volume. As suggested by normal tissue complication probability modeling for radiosurgery,^(^ ^1^ ^)^ a high degree of conformity of the prescription dose to target volume should be achieved to allow safe treatment of the target. Conformity Index (CI) is the ratio of the prescription volume to the target volume, as defined in the Radiation Therapy Oncology Group (RTOG) radiosurgery guidelines in 1993.^(^ ^2^ ^–^ ^4^ ^)^ CI is useful for evaluating competing plans for the same patient or comparing different modalities. Dose volume histograms (DVH) summarize the dose distribution information for a region of interest or anatomical structure and identify characteristics such as dose uniformity and hot or cold spots. DVHs may be a preliminary step in evaluating statistics such as tumor control and normal tissue complication probabilities.^(^ ^5^ ^,^ ^6^ ^)^ However, when comparing a large number of plans, DVHs contain large amounts of data and make the comparison difficult and cumbersome.

Radiosurgery has evolved over the past decade with the development of new treatment delivery technologies such as dedicated radiosurgery linear accelerators. In the past, it has been reported that linear accelerator‐based SRS is less conformal than gamma knife SRS.^(^ ^4^ ^,^ ^7^ ^,^ ^8^ ^)^ However, linear accelerator‐based SRS has become highly sophisticated, evolving from circular arc and multiple isocenters per target to dynamic conformal arcs (DCA) based on a single isocenter. The dynamic conformal arc is a method of linear accelerator‐based SRS that uses multiple arcs rotating about a single isocenter.^(^ ^9^ ^,^ ^10^ ^)^ This method uses the MLC to conform to the target volume every 10 degrees of arc. Recently, it has been reported that improvement in linear accelerator‐based SRS techniques have allowed comparable conformity to that of a gamma knife.^(^ ^11^ ^)^

The impacts of linear accelerator MLC leaf width on stereotactic radiosurgery and radiotherapy plans have been investigated and previously reported.^(^ ^12^ ^–^ ^18^ ^)^ Kubo et al.^(^ ^12^ ^)^ compared the conformity of 3D conformal plans using 1.7 mm, 3 mm and 10 mm leaf width MLCs and found that the smaller leafs produced more normal tissue sparing. IMRT plans for cranial cases were compared using 5 mm and 10 mm MLC leaf widths, and noticeably better sparing of optic structure was observed using 5 mm MLC.^(^ ^13^ ^)^ Monk et al.^(^ ^14^ ^)^ compared the Varian Millennium 120‐MLC (minimal 5 mm leaf) with the BrainLAB (BrainLAB, Munich, Germany) micro‐MLC (minimal 3 mm leaf width) with plans using fixed non‐coplanar beams. They reported a small but statistically significant improvement in dose conformity and organ at risk (OAR) sparing with the 3 mm MLC compared with the 5 mm MLCs. Jin et al.^(^ ^17^ ^)^ used dynamic conformal arcs and IMRS/IMRT techniques to compare the 3 mm micro‐MLC and the 5 mm and 10 mm MLC, and found significant dosimetric differences in the conformity indices between the three MLCs – with the 3 mm leaf width scoring better. This study also reported that the difference in the conformity index decreases with the target volume and that, as the MLC margin increases in increments of 1 mm, the difference in the conformity indices decreases. Chern et al.^(^ ^18^ ^)^ also compared the 3 mm BrainLAB micro‐MLC (minimal leaf width is 3 mm) and Varian Millennium 120‐MLC (minimal leaf width is 5 mm) using the DCA technique. This paper reported improved dosimetric results using 3 mm as compared to 5‐MLC and for small target volume ($<1\;{\text{cm}}^{3}$), they reported as high as 10% improvement, on average, in CI.

Our current study compares the dose distributions between a high‐definition 2.5 mm MLC leaf width (${\text{MLC}}_{2.5}$) (High Definition (HD) MLC, Varian Medical Systems, Palo Alto, CA) and a standard 5 mm MLC leaf width (${\text{MLC}}_{5}$) (Millennium MLC, Varian Medical Systems, Palo Alto, CA) in the treatment of intracranial lesion using DCA treatment techniques. Clinical patient cases were planned with both MLCs using the same DCA beam paths. Figures of merit (FOM) for plan comparison such as CI, target volume dose coverage, and normal tissue sparing were used to evaluate the superiority of the resulting treatment plans. The effect of target volume on the performance of the two MLCs also was analyzed using simulated and actual patient case data.

## II. MATERIALS AND METHODS

### A. Treatment delivery MLCs

A Novalis Tx (Varian Medical Systems, Palo Alto, CA) radiosurgical linear accelerator with a high definition (HD) MLC is one machine used by this study. This linear accelerator has 6 MV photon beam energy with a 1000 MU/min maximum dose rate. The Novalis Tx has a maximum possible MLC field size of $22\times 40\;{\text{cm}}^{2}$ at isocenter. The 22 cm is formed by 32 leaf pairs of 2.5 mm leaf width in the central part and 28 leaf pairs of 5 mm leaf width in the outer part of the MLC. This HD MLC is referred to as the ${\text{MLC}}_{2.5}$ in this paper. A Trilogy (Varian Medical Systems, Palo Alto, CA) linear accelerator using a standard 120‐leaf MLC is used as the comparison system in this study. The Trilogy has a 6 MV and 18 MV photon beam energy with a maximum dose rate of 1000 MU/min. The 120‐leaf MLC has two leaf widths with the inner 40 leaf pairs having a 5 mm leaf width and the outer 20 leaf pairs having 10 mm leaf width. The maximum field size for the standard120‐leaf MLC is $40\times 40\;{\text{cm}}^{2}$. This standard Trilogy MLC is referred to as the ${\text{MLC}}_{5}$ in this paper. All target volumes in this study were small and utilized the inner set of leaves to enable a direct comparison of 2.5 mm and 5 mm leaf widths.

### B. Simulated target volume

A simulated patient case was created to study solely the effect of target volume size and shape on the performance of the two MLC systems. For an example patient case, a sphere was created as the target volume with volumes ranging from 0.11 to $39\;{\text{cm}}^{3}$. The simulated sphere was centrally located within the patient head.

### C. Study patients

A total of 43 stereotactic radiosurgery patients were selected for this study. These patients were selected from 278 patients treated since January 2007 in our institution. All of these (except one patient treated with IMRS) were treated with DCA method for SRS. Patient selection was based on including a wide variety of shapes and volumes of radiosurgery targets. The selected cases included 11 patients with acoustic neuromas, 3 with meningiomas, 4 with arteriovenous malformations, 19 with metastatic lesions, 3 with glomus tumors, 1 with an astrocytoma, 1 with a pineocytoma and 1 with a pituitary adenoma. The patients' ages ranged from 26 to 81 yrs old, with an average age of 55 yrs. The volume of targets ranged from 0.07 to $40.57\;{\text{cm}}^{3}$ and the average volume was $5.9\;{\text{cm}}^{3}$. All these patients were planned using DCA technique for both ${\text{MLC}}_{2.5}$ and ${\text{MLC}}_{5}$.

### D. Treatment plan creation

**Figure 1:**
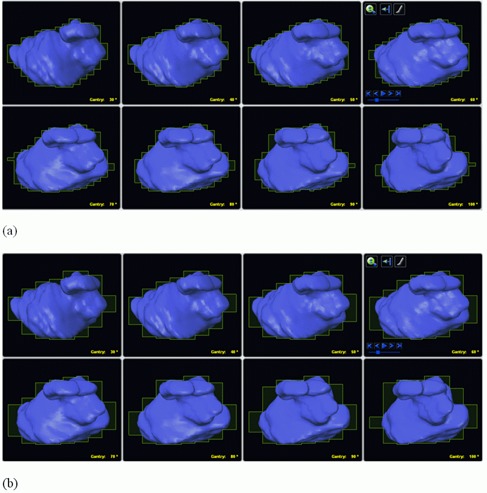
Graphical display of BEV for 100° arc for ${\text{MLC}}_{2.5}$ (a) and ${\text{MLC}}_{5}$ (b). These two views show MLC's leaf position for every ten degrees surrounding the target volume (in blue). The leaf width between the two views for the 2.5 mm and 5 mm leaf width is shown; the yellow outline demonstrates the difference in conformality of the two MLC systems.

The DCA technique was used for all the plans in this study. In this technique, the MLC automatically conforms to the target volume outline via software methods. Figure 1 shows the difference via beam's eye view (BEV) between the ${\text{MLC}}_{2.5}$ and ${\text{MLC}}_{5}$ conforming to a target volume. The treatment plans were created using a dedicated radiosurgical planning system (iPlan RT Dose 3.0, BrainLAB, Germany). A planning CT image study was obtained for each patient. Patient images were acquired with a stereotactic localization frame (BrainLAB, Germany) attached to the patient's head. The CT imaging slice thickness was 0.625 mm. Magnetic resonance (MR) images were carried out for each patient at a similar slice thickness. The CT and MR were registered together to allow for MR‐based target delineation. Target volumes and critical structures were outlined by our radiation oncologist and neurosurgeon during planning process. A DCA plan was created using 4 or 5 non‐coplanar dynamic conformal arcs averaging 100° per arc. The treatment planning system using the MLC automatically creates a field shape that conforms to the target outlines as shown in Fig. 1. The collimator angles were set to 90° and, in few cases, it was changed to improve MLC conformity with the target volume. For non‐metastatic lesions, no margin was added to create a planning target volume (PTV). For all metastatic lesions, a margin of 1 mm was added to create a PTV. To compare the ${\text{MLC}}_{2.5}$ and ${\text{MLC}}_{5}$ plans, all treatment parameters were identical for planning purposes.

### E. Treatment plan evaluation

The evaluation of competing MLC‐based plans was performed using isodose displays with medical images, DVHs, and figures of merits (FOM). The use of isodose displays and DVHs are standard plan evaluation tools used in the clinical environment. Dose volume histograms (DVHs) were generated for each lesion and for the surrounding normal brain tissue. In this analysis, the use of FOM allows for a rapid and objective assessment on the quality of the two treatment plans. This section describes the FOMs used for evaluating the plan differences in this study.

#### E.1 Target coverage

Target coverage (TC) is defined as the percent volume of the tumor volume receiving the prescription dose. Typically, the coverage index should be at least 95%. In this study, the prescription isodose surface was selected as the greatest isodose surface covering ≥95% of the target volume and delivering 95% of the prescription dose to 99% of target volume.^(^ ^11^ ^,^ ^18^ ^)^ After the prescription isodose surface was determined for the ${\text{MLC}}_{2.5}$, the equivalent isodose surface was used for the ${\text{MLC}}_{5}$. Table 1 shows data for an example patient case with a target volume of $4.93\;{\text{cm}}^{3}$. Using this example data in Table 1, the 88% isodose surface covers 95.5% of the target volume, which satisfies the first condition. The second condition is also satisfied since 95% of this prescription isodose value (88%×95%=83.6%) covers 99% of the target volume. In this analysis, if the first condition was fulfilled and the second condition was not fulfilled, then a lower isodose surface was selected until both conditions are satisfied.

**Table 1:** An example patient case demonstrating prescription isodose selection method for study (target volume for this case = $4.93\;{\text{cm}}^{3}$).

*PI* a	*Target Coverage (%)*	*95% of PI*	*CI*	*PITV Ratio*
79	100.00	75.05	2.293	2.293
80	100.00	76.00	2.209	2.209
81	99.94	76.95	2.162	2.161
82	99.89	77.90	2.083	2.081
83	99.72	78.85	2.013	2.007
84	99.50	79.80	1.951	1.941
85	98.94	80.75	1.871	1.852
86	98.05	81.70	1.798	1.763
87	97.00	82.65	1.720	1.669
88	95.55	83.60	1.656	1.582
89	93.49	84.55	1.615	1.510
90	90.60	85.50	1.590	1.441
91	87.09	86.45	1.500	1.306
92	84.09	87.40	1.427	1.200
93	79.13	88.35	1.368	1.083
94	73.79	89.30	1.276	0.941
95	66.78	90.25	1.209	0.807
96	57.93	91.20	1.165	0.675
97	47.30	92.15	1.118	0.529
98	34.61	93.10	1.092	0.378
99	19.76	94.05	1.061	0.210

a Prescription isodose value

#### E.2 Normal tissue sparing

To evaluate the normal tissue sparing associated with the MLC systems, an anatomical structure consisting of an adjacent tissue shell was created to surround the target volume by adding a 1 cm margin. This is similar to the method described by Chern et al.^(^ ^18^ ^)^ The DVH for this normal tissue structure was computed to assess the dose volume values for selected points on the DVH curve. The normal tissue volume 50 (${\text{NTV}}_{50}$) was calculated which is the normal tissue receiving 50% of the prescription isodose (PI). The ${\text{NTV}}_{70}$ and ${\text{NTV}}_{90}$ receiving 70% and 90% of prescription isodose, respectively, were also computed from the DVH.

**Figure 2:**
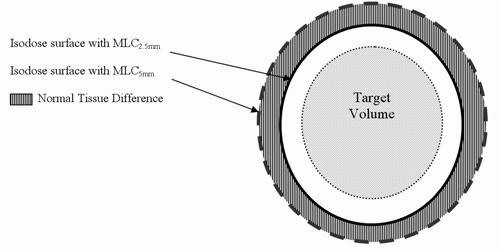
Schematic illustrating the definition of normal tissue difference NTD.

To assess normal tissue sparing, a normal tissue difference (NTD) was calculated. NTD is the difference between the volume of normal tissue receiving a certain dose utilizing ${\text{MLC}}_{5}$ and the volume receiving the same dose using ${\text{MLC}}_{2.5}$. This is illustrated in Fig. 2 with a schematic diagram showing the striped region as the area calculated for the NTD. For example, the NTD for tissue receiving 50% of the prescription isodose (${\text{NTD}}_{50}$) is calculated as follows:

(1) $$NTD_{50}=NTV_{50}^{MLC_{5mm}}-NTV_{50}^{MLC_{2.5mm}}$$

where $NTV_{50}^{MLC_{5mm}}$ and $NTV_{50}^{MLC_{2.5mm}}$ are the volume of normal tissue receiving 50% of PI with the use of ${\text{MLC}}_{5}$ and ${\text{MLC}}_{2.5}$, respectively.

#### E.3 Target conformity

Conformity indices are used to compare competing plans, evaluate treatment technique, and assess clinical complications by quantifying the dose conformity to a target volume. Several different indices have been reported to evaluate the conformity of prescription isodose surface to the target volume. In 1993, Radiation Therapy Oncology Group (RTOG) proposed routine evaluation of stereotactic radiotherapy treatment plans based on reference isodose value and reference isodose volume and target volume.^(^ ^2^ ^–^ ^4^ ^,^ ^7^ ^,^ ^19^ ^)^ The RTOG proposed the conformity index as PITV^(^ ^4^ ^,^ ^7^ ^,^ ^8^ ^,^ ^11^ ^,^ ^19^ ^,^ ^20^ ^)^ which is defined as:

(2) $$PITV=\frac{PIV}{TV}$$

where TV is the target volume and PIV is the prescription isodose volume.

The RTOG Conformity index, or *PITV* ratio, is the most frequently used conformity index to compare the conformity of treatment plans from different radiosurgery delivery systems.^(^ ^4^ ^,^ ^7^ ^,^ ^8^ ^,^ ^21^ ^–^ ^24^ ^)^ According to the *PITV* ratio, a PITV value equal to 1 corresponds to ideal conformation. A PITV greater than 1 indicates that the irradiated volume is greater than the target volume, which means it includes healthy tissues. If the PITV is less than 1, it indicates that the target volume is partially covered. According to RTOG guidelines, a PITV^(^ ^19^ ^)^ between 1 and 2 is considered a treatment plan of acceptable dose conformity. The treatment plan receives a minor violation judged by RTOG guidelines for a PITV between 2 and 2.5 or 0.9 and 1. The treatment plan is rated a major violation by RTOG standards if the PITV is less than 0.9 or more than 2.5.

The drawback of the *PITV* ratio is that it does not take into account the spatial location and the shape of the prescription isodose volume relative to the TV. If the volume of tissue receiving the prescribed dose is equal to the TV, the *PITV* ratio will be 1 and the treatment plan will receive the same perfect score of 1 regardless whether the prescribed isodose perfectly enclosed the TV or completely missed the TV (i.e. 0% of the TV received the prescribed dose). Therefore, *PITV* ratio can be improved by accounting for coverage of the target volume.

Paddick^(^ ^20^ ^)^ accounted for target volume coverage by proposing a new conformity index as follows:

(3) $$CI_{Paddick}=\frac{TV_{PIV}}{TV}\times \frac{TV_{PIV}}{PIV}$$

where ${\mathrm{TV}}_{\mathrm{PIV}}$ is the target volume within the prescribed isodose volume *PIV*. This becomes the inverse of the PTIV when the prescription isodose fully covers the target volume.

Paddick's Conformity CI was modified by Nakamura et al.^(^ ^7^ ^)^ and expressed as follows:

(4) $$CI=\frac{PIV/PVTV}{PVTV/TV}$$

where is the target volume, *PIV* is the prescription isodose volume, *PVTV* is the *TV* included in the prescription isodose surface. The *PIV* equals to the *PVTV* plus the normal tissue NT encompassed by the prescription isodose surface. The numerator of the above equation measures the excess volume of normal tissue within the prescription isodose surface and the denominator measures the target coverage. In this study, we used a similar conformity index method, which is as follows:

(5) $$CI=\frac{PIV}{PVTV}=\frac{\left(PVTV+NT_{V}\right)}{PVTV}$$

where *PIV* is the total volume encompassed by the prescription isodose surface, *PVTV* is the volume of target TV encompassed by the prescription isodose surface, ${\mathrm{NT}}_{V}$ is the volume of the normal tissue encompassed by the prescription isodose surface. It has been demonstrated that prescription isodose surface associated with the minimal CI does not necessarily produce provide adequate coverage. This is due to the fact that, as the coverage of the TV increases, a large amount of NT is included in the PIV and the plan will have higher CI. Therefore, the prescription isodose surface should be chosen that balances conformity and target coverage.

In another report, Paddick et al.^(^ ^25^ ^)^ also proposed a dose gradient index (GI), which is the ratio of the volume of 50% of the prescription isodose to the volume of the prescription isodose to compare plans of equal conformity indices. The GI shows which plan gives the steepest dose falloff outside the target. Other investigators introduced a conformity distance index (CDI).^(^ ^26^ ^)^ The CDI is defined as the average distance between the prescription isodose and the target contour. This parameter accounts for the influence of target size and shape complexity on the conformity of the plan. The CDI is expressed as follows:

(6) $$CDI=\frac{\left(PIV-TV_{PIV})+(TV-TV_{PIV}\right)}{\frac{1}{2}\times (S_{PIV}+S_{TV})}$$

where $S_{\mathrm{PIV}}$ and $S_{\mathrm{TV}}$ are the surfaces of ${\mathrm{TV}}_{\mathrm{PIV}}$ and *PIV*, respectively. The CDI values were accurately calculated using custom‐developed software by the authors.

## III. RESULTS

### A. Simulated target study

**Figure 3:**
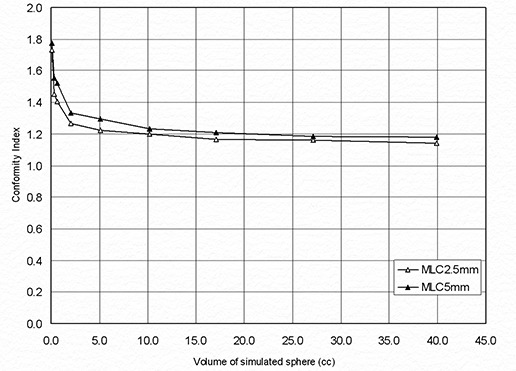
Dependence of Conformity Index on target volume for a simulated spherical target using ${\text{MLC}}_{2.5}$ and ${\text{MLC}}_{5}$.

**Figure 4:**
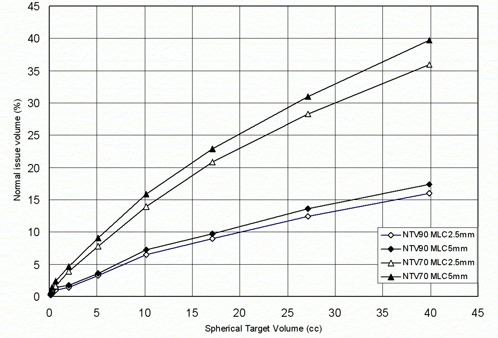
Comparison of normal tissue sparing capabilities of ${\text{MLC}}_{2.5}$ and ${\text{MLC}}_{5}$ for a simulated spherical target.

In our study, simulated spherical target volumes (ranging from 0.11 to $39\;{\text{cm}}^{3}$) were analyzed. These targets were created to reduce target shape effects on the CI and evaluate its dependence on the target volume only, for both MLCs. The plotted results show that, while CIs for both MLCs decrease with the target volume, the CI for ${\text{MLC}}_{2.5}$ for each TV volume is clearly lower than the corresponding CI of ${\text{MLC}}_{5}$ across the entire range of volumes (Fig. 3). Figure 4 demonstrates that ${\text{NTV}}_{70}$ and ${\text{NTV}}_{90}$ also increase with the volume of a spherical target using both MLC and that the ${\text{MLC}}_{2.5}$ provides better normal tissue sparing.

### B. Study patients

**Figure 5:**
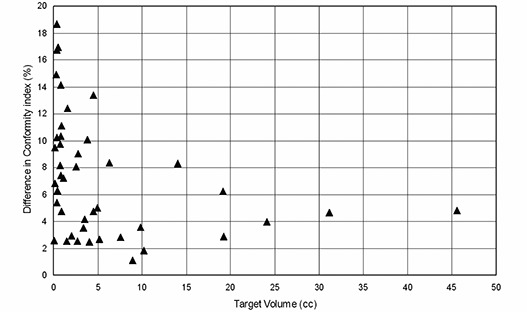
The percentage difference of the conformity indices between ${\text{MLC}}_{5}$ and ${\text{MLC}}_{2.5}$ versus target volume.

Table 2 presents the ${\text{MLC}}_{2.5}$ conformity indices which range from 1.15 to 2.44 with a median of 1.59. The CI range for ${\text{MLC}}_{5}$ was 1.60 to 2.85 with a median of 1.71. Similar to our results with the simulated target study, these results indicate that the ${\text{MLC}}_{2.5}$ provides better treatment conformity than ${\text{MLC}}_{5}$ as judged by the CI values in a range of actual patient tumors. The graph of percentage difference of the conformity indices between ${\text{MLC}}_{5}$ and ${\text{MLC}}_{2.5}$ versus target volume is shown (Fig. 5). While differences in CIs between the two MLCs decrease with target volume, even at large volumes the ${\text{MLC}}_{2.5}$ provides better conformity.

**Table 2:** Tumor type, volume and comparison of conformity index between ${\text{MLC}}_{2.5}$ and ${\text{MLC}}_{5}$ for each patient.

*Case #*	*Tumor Type*	*Age*	*Gender*	*TV (cm^3^)*	*CI‐* ${\mathrm{MLC}}_{2.5}$	*CI‐* ${\mathrm{MLC}}_{5}$	*% Diff. CI*
1	Acoustic Neuroma	46	Female	0.070	1.888	1.936	2.560
2	Acoustic Neuroma	68	Male	0.187	1.902	2.083	9.474
3	Acoustic Neuroma	40	Female	0.327	2.367	2.720	14.920
4	Acoustic Neuroma	53	Female	0.351	2.081	2.470	18.670
5	Acoustic Neuroma	79	Female	0.382	1.950	2.150	10.230
6	Acoustic Neuroma	68	Female	0.411	2.441	2.849	16.736
7	Acoustic Neuroma	59	Male	0.505	1.993	2.331	16.958
8	Acoustic Neuroma	49	Male	0.823	1.992	2.198	10.314
9	Acoustic Neuroma	37	Male	0.836	1.421	1.622	14.147
10	Acoustic Neuroma	59	Male	0.867	1.563	1.637	4.722
11	Acoustic Neuroma	81	Female	1.103	1.619	1.735	7.188
12	AVM^a^	35	Male	1.612	1.861	2.092	12.401
13	AVM	35	Female	3.803	2.174	2.393	10.066
14	AVM	58	Female	4.532	1.988	2.254	13.390
15	AVM	26	Female	6.289	1.920	2.080	8.351
16	Glomus Tumor	66	Female	3.537	1.679	1.749	4.136
17	Glomus Tumor	39	Female	2.723	1.411	1.447	2.495
18	Glomus Tumor	55	Female	4.525	1.484	1.555	4.764
19	Pilocytic Astrocytomas	50	Female	2.811	1.412	1.539	9.013
20	Pituitary Adenoma	48	Female	2.040	1.400	1.441	2.934
21	Pineocytoma	38	Male	9.847	1.243	1.287	3.542
22	Meningioma	60	Female	0.715	1.652	1.787	8.136
23	Meningioma	45	Male	5.170	1.498	1.539	2.689
24	Meningioma	63	Female	8.942	1.147	1.160	1.102
25	Metastatic Lesion	50	Male	0.168	1.354	1.447	6.823
26	Metastatic Lesion	76	Female	0.357	1.523	1.605	5.379
27	Metastatic lesion	71	Male	0.449	1.495	1.589	6.274
28	Metastatic Lesion	54	Male	0.782	1.306	1.433	9.715
29	Metastatic Lesion	53	Female	0.825	1.310	1.407	7.397
30	Metastatic Lesion	59	Female	0.864	1.519	1.688	11.129
31	Metastatic Lesion	63	Female	1.530	1.241	1.272	2.528
32	Metastatic Lesion	34	Female	2.583	1.421	1.536	8.069
33	Metastatic Lesion	80	Female	3.370	1.252	1.296	3.508
34	Metastatic Lesion	63	Female	4.019	1.239	1.269	2.455
35	Metastatic Lesion	81	Female	4.931	1.656	1.738	4.983
36	Metastatic Lesion	62	Female	7.602	1.541	1.584	2.817
37	Metastatic Lesion	37	Male	10.187	1.153	1.174	1.838
38	Metastatic Lesion	55	Female	14.024	1.382	1.497	8.319
39	Metastatic Lesion	68	Male	19.112	1.194	1.268	6.204
40	Metastatic Lesion	48	Male	19.240	1.471	1.513	2.843
41	Metastatic Lesion	48	Male	24.136	1.430	1.487	3.963
42	Metastatic Lesion	48	Male	31.128	1.403	1.468	4.624
43	Metastatic Lesion	48	Male	45.576	1.317	1.380	4.798

a Arteriovenous malformation

**Figure 6:**
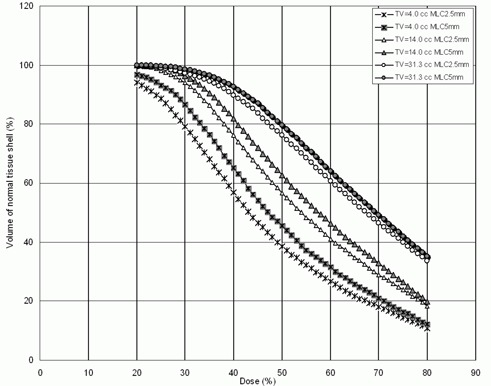
Dose volume histograms of the normal tissue shell surrounding target for three patients.

**Figure 7:**
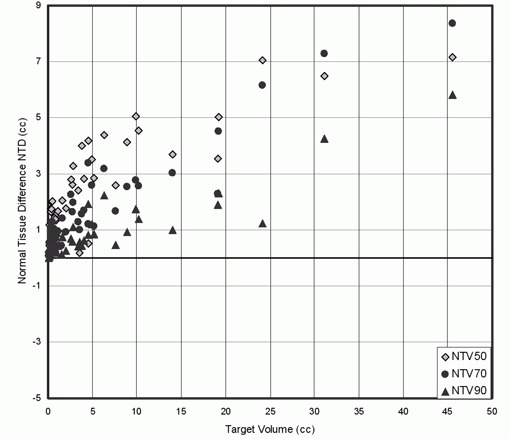
Normal tissue difference NTD plotted as a function of target volume for tissue irradiated to 50%, 70% and 90% when ${\text{MLC}}_{2.5}$ is used instead of ${\text{MLC}}_{5}$.

Figure 6 shows dose volume histograms of the 1 cm normal tissue shell surrounding representative target volumes. In this figure, the normal tissue volume receiving doses between 20–80% of the prescription dose is reduced when ${\text{MLC}}_{2.5}$ is used. Comparison of the normal tissue sparing capabilities of the ${\text{MLC}}_{2.5}$ and ${\text{MLC}}_{5}$ is shown in Fig. 7 and Table 3. As illustrated by this data, ${\text{NTV}}_{50}$, ${\text{NTV}}_{70}$ and ${\text{NTV}}_{90}$ for ${\text{MLC}}_{2.5}$ plans were reduced significantly, resulting in positive NTD values in each case.

**Table 3:** Conformity distance index (CDI) and normal tissue difference (NTD) between ${\text{MLC}}_{2.5}$ and ${\text{MLC}}_{5}$ in cm^3^ of volume receiving 50%, 70% and 90% of the prescription isodose.

*Case #*	$TV(cm^{3})$	$NTD_{50}(cm^{3})$	$NTD_{70}(cm^{3})$	$NTD_{90}(cm^{3})$	${\mathrm{CDI‐MLC}}_{2.5}(mm)$	$CDI‐MLC_{5}(mm)$
1	0.070	0.210	0.071	0.007	1.41	1.87
2	0.187	1.167	0.547	0.148	1.39	1.65
3	0.327	1.632	0.889	0.349	1.88	2.35
4	0.351	1.765	0.950	0.350	1.83	2.38
5	0.382	1.743	1.103	0.560	1.55	1.75
6	0.411	1.992	1.232	0.488	1.96	2.25
7	0.505	2.034	1.293	0.560	1.84	2.35
8	0.823	1.543	1.009	0.484	1.83	2.13
9	0.836	1.504	0.988	0.356	1.18	2.05
10	0.867	1.344	0.752	0.224	1.16	1.85
11	1.103	1.660	0.951	0.429	1.85	2.29
12	1.612	2.052	1.412	0.732	3.33	3.87
13	3.803	4.004	1.568	0.432	3.53	4.14
14	4.532	4.182	3.374	1.919	3.61	4.04
15	6.289	4.388	3.192	2.224	4.30	6.15
16	3.537	0.168	1.005	0.560	2.06	2.29
17	2.723	2.624	1.640	0.584	1.57	1.59
18	4.525	0.504	1.204	0.812	2.29	2.96
19	2.811	3.273	1.965	1.110	2.10	2.85
20	2.040	1.764	0.932	0.246	1.63	1.97
21	9.847	5.064	2.768	1.744	2.08	2.88
22	0.715	1.448	0.679	0.233	1.66	1.78
23	5.170	2.848	1.132	0.836	1.96	2.28
24	8.942	4.136	2.528	0.912	1.79	2.29
25	0.168	0.448	0.203	0.055	1.98	2.31
26	0.357	1.254	0.752	0.213	0.96	1.37
27	0.449	0.916	0.504	0.210	0.92	1.45
28	0.782	1.388	0.782	0.301	1.86	2.14
29	0.825	1.024	0.660	0.288	1.26	1.65
30	0.864	0.616	0.376	0.176	1.91	1.99
31	1.530	0.836	0.432	0.120	1.92	2.26
32	2.583	2.788	2.252	0.696	1.65	2.49
33	3.370	2.400	1.276	0.416	1.37	2.84
34	4.019	2.832	1.680	0.608	1.32	1.46
35	4.931	3.523	2.598	1.211	2.56	2.81
36	7.602	2.584	1.672	0.456	2.92	3.52
37	10.187	4.530	2.556	1.390	1.62	1.77
38	14.024	3.680	3.032	0.996	3.47	4.37
39	19.112	3.548	2.284	1.888	3.21	3.69
40	19.240	5.024	4.520	2.296	3.23	3.79
41	24.136	7.056	6.160	1.240	3.72	4.54
42	31.128	6.488	7.288	4.248	4.53	5.24
43	45.576	7.161	8.360	5.832	4.84	5.47

Table 3 also shows the values of CDI for all cases using both MLCs. The values of this parameter ranged were 0.9 to 4.8 mm for ${\text{MLC}}_{2.5}$ and from 1.37 to 6.15 mm for ${\text{MLC}}_{5}$. The values of CDI indicate that the ${\text{MLC}}_{2.5}$ is more suitable than ${\text{MLC}}_{5}$ for highly complex shaped targets.

## IV. DISCUSSION

Our study shows that the dose conformity of the ${\text{MLC}}_{2.5}$ is significantly better than ${\text{MLC}}_{5}$. For target volumes ranging from 0.07 to $45.6\;{\text{cm}}^{3}$, the ${\text{MLC}}_{2.5}$ CI improved by an average of 7.3%. For small targets such as acoustic nueroma ($<1.1\;{\text{cm}}^{3}$), an average CI improvement was 11% for ${\text{MLC}}_{2.5}$ plans. For our cohort of patients studied (see Table 2), the CI was always better when ${\text{MLC}}_{2.5}$ was used, with the greatest CI difference of 18% seen for a lesion of $0.35\;{\text{cm}}^{3}$.

Our study showed that the overall average difference in the CI between the two MLCs increases from 4.6% for the simulated spherical cases to 7.3% for the patients in this study. Since the shape of a spherical target is simple and targets of actual patients are more complex, these results suggest that ${\text{MLC}}_{2.5}$ yields better conformity for complex tumor shape than ${\text{MLC}}_{5}$.

Our results showed that the CDI values for all tumor sizes and shapes studied were less when ${\text{MLC}}_{2.5}$ was used compared to those with ${\text{MLC}}_{5}$. The CDI values were higher for highly complex targets (such as in case 16 which is an AVM) and low for uniform shapes (such as in case 27) as shown in Table 3.

The ${\text{MLC}}_{2.5}$ is a better choice to treat SRS target volume with DCA treatment technique particularly for small lesions and geometrically complex tumors. Other investigators have reported modest but statistically significant improvements using small leaf MLCs.^(^ ^9^ ^,^ ^11^ ^,^ ^13^ ^,^ ^18^ ^)^ In particular, investigators from other institutions compared 3 mm leaf width MLC (${\text{MLC}}_{3\text{mm}}$) with ${\text{MLC}}_{5}$ and reported better target conformity and tissue sparing with the ${\text{MLC}}_{3\text{mm}}$.^(^ ^14^ ^,^ ^17^ ^,^ ^18^ ^)^ Hazard et al.^(^ ^11^ ^)^ compared linear accelerator‐based SRS using DCA with a Gamma Knife system and reported that accelerator‐based SRS provided comparable treatment conformity. Thus, ${\text{MLC}}_{2.5}$ linear accelerator‐based SRS system should offer at least equivalent conformity when compared with Gamma Knife systems. With both simulated targets and actual patient tumors, the difference in the conformity indices between ${\text{MLC}}_{5}$ and ${\text{MLC}}_{2.5}$ decreased with target volume (Fig. 3 and 5). Two separate studies reported in the literature^(^ ^17^ ^,^ ^18^ ^)^ found similar trends, whereas Monk et al.^(^ ^14^ ^)^ did not report any dependence of CI on target volume.

Figures 4, 6 and 7 show that using ${\text{MLC}}_{2.5}$ improves normal tissue sparing. Figure 7 shows that NTD increased with target volume and its values were always positive, suggesting better normal tissue sparing with the use of ${\text{MLC}}_{2.5}$ compared to ${\text{MLC}}_{5}$. Based on our analysis, ${\text{MLC}}_{2.5}$ provides better normal tissue sparing than ${\text{MLC}}_{5}$ for intracranial lesions even where the CI difference is modest. This is significant for a patient where the tumor is located close to the brainstem or the optic chiasm. In this scenario, the ${\text{MLC}}_{2.5}$ could offer a conformal therapeutic dose to the target volume while providing a lower dose for the adjacent critical structure. For such a patient, the dose to critical structure could prohibit the treatment using the ${\text{MLC}}_{5}$.

## V. CONCLUSIONS

The ${\text{MLC}}_{2.5}$ has a dosimetric advantage over the ${\text{MLC}}_{5}$ both in the treatment conformity (CI and CDI) and normal tissue sparing when target shape complexity increases. The ${\text{MLC}}_{2.5}$ has an advantage particularly when the critical structures are adjacent to the target volume. The use of ${\text{MLC}}_{2.5}$ improved the plan conformity for small lesions ($<1.1\;{\text{cm}}^{3}$) on average by 11%, as compared to the ${\text{MLC}}_{5}$. In summary, the ${\text{MLC}}_{2.5}$ has the potential to provide improved dose conformity to the target volume and lower doses to critical structures compared with standard MLCs such as ${\text{MLC}}_{5}$. The 2.5 mm leaf width for an MLC represents a new standard for linear accelerator‐based radiosurgery.
